# Characterization of the Partitioning System of *Myxococcus* Plasmid pMF1

**DOI:** 10.1371/journal.pone.0028122

**Published:** 2011-12-09

**Authors:** Xia Sun, Xiao-jing Chen, Jing Feng, Jing-yi Zhao, Yue-zhong Li

**Affiliations:** State Key Laboratory of Microbial Technology, School of Life Science, Shandong University, Jinan, China; Baylor College of Medicine, United States of America

## Abstract

pMF1 is the only autonomously replicating plasmid that has been recently identified in myxobacteria. This study characterized the partitioning (*par*) system of this plasmid. The fragment that significantly increased the retaining stability of plasmids in *Myxococcus* cells in the absence of selective antibiotics contained three open reading frames (ORFs) *pMF1.21*-*pMF1.23* (*parCAB*). The *pMF1.22* ORF (*parA*) is homologous to members of the *parA* ATPase family, with the highest similarity (56%) to the *Sphingobium japonicum* ParA-like protein, while the other two ORFs had no homologs in GenBank. DNase I footprinting and electrophoretic mobility shift assays showed that the *pMF1.23* (*parB*) product is a DNA-binding protein of iteron DNA sequences, while the product of *pMF1.21* (*parC*) has no binding activity but is able to enhance the DNA-binding activity of ParB to iterons. The ParB protein autogenously repressed the expression of the *par* genes, consistent with the type Ib *par* pattern, while the ParC protein has less repressive activity. The ParB-binding iteron sequences are distributed not only near the partitioning gene loci but also along pMF1. These results indicate that the pMF1 *par* system has novel structural and functional characteristics.

## Introduction

Myxobacteria are well known for their unique multicellular social behavior [1] and the production of diverse and novel bioactive secondary metabolites [2]. However, autonomously replicating plasmids have not been reported until recently. In 2008, we reported the discovery of the first and as yet only endogenous myxobacterial plasmid, pMF1, which was isolated from *Myxococcus fulvus* strain 124B02 [3]. Except for a few ORFs that are highly homologous to those in myxobacterial genomes, most ORFs in the *Myxococcus* plasmid pMF1 do not have homologs in the GenBank database, including the replication-associated sequence. We localized the replication region to the *pMF1.14* ORF using a vector that cannot replicate in *M. xanthus*, and shuttle vectors between *Escherichia coli* and *M. xanthus* were thus successfully constructed [3]. Because they lack a stabilizing sequence, these low-copy-number shuttle vectors are not stably inherited in *M. xanthus* and are easily lost in the absence of selective antibiotics.

Low-copy-number plasmids are dependent on plasmid-encoded partitioning (*par*) systems for stable segregation into daughter cells [4]. The *par* loci of a plasmid typically consist of an operon containing an autogenously regulated gene pair, *parA* and *parB* [5], [6], [7], [8], [9], and one or more centromere-like iteron regions [5], [10], [11]. ParA is an ATPase (other two classes of motor proteins are GTPases ParM and TubZ) involved in the segregation of plasmids or chromosomes [12], [13], [14], [15], while ParB is a DNA-binding protein that recognizes specific iteron sequences of the centromere-like sites that are normally located upstream or downstream in the *par* loci [16], [17], [18]. In general, the two plasmid-encoded *trans*-acting partitioning proteins and the *cis-*acting centromeric site are all essential for stable segregation of plasmid. The plasmid partitioning systems have been identified into three types. The type I *par* loci encode an ATPase containing Walker motifs, while types II and III encode actin-like and tubulin-like proteins, respectively [4], [19]. Type I *par* system is further divided into two sub-groups, designated type Ia and type Ib, based on the regulation manner of the operon, the properties of ParB, and the location of the *cis*-acting sites [4]. In the type Ia *par* system, represented by P1 and F plasmids, the sizes of ParA and ParB proteins are 321–420aa and 312–342aa, respectively [4]. The Ia ParA contains an N-terminal DNA–binding domain, which plays an autoregulation role for the transcription of operon. In contrast to the type Ia *par* system, the type Ib, represented by pTAR, TP228, pB171 and pSM19035, encodes a ParA protein of 192–308aa, lacking the N-terminal DNA–binding domain [4], [20], [21], [22], [23]. The ParB protein of this sub-group is 46–131aa in size, and is able to bind to the *par* operon promoter and regulates the transcription of the operon [4].

In this work, the pMF1 partitioning system region was screened using pZJY41, a shuttle plasmid containing the replication region of pMF1, and was able to replicate in *Myxococcus* cells. The partitioning system, containing three ORFs, *pMF1.21*-*pMF1.23*, was characterized. The results showed that the pMF1 partitioning system has novel characteristics with respect to its special organization, functioning patterns and dispersed distribution of the *cis-*acting centromeric sequences.

## Results

### Isolation of the partition-associated region of plasmid pMF1

The shuttle vector pZJY41 can replicate in *M. xanthus* DZ1 but contains no additional stabilizing sequence, and is thus easily lost in *Myxococcus* cells during the subculture in the absence of selective antibiotics [3]. To screen the partitioning system region of pMF1, we established a small library of pMF1 fragments, and inserted these fragments into the pZJY41 vector, respectively (Table 1). Unpublished data suggest that the shuttle plasmid pZJY41 is sometimes structurally unstable in *Myxococcus* cells when a fragment that is larger than 8 kb is inserted. Thus, the pMF1 insertion fragments were all between 1.5 kb and 8 kb. The constructed plasmids were transformed into *M. xanthus* DZ1. Electrophoresis results demonstrated that none of the plasmids became smaller, suggesting that they were structurally stable in DZ1.

**Table 1:** Bacterial strains and plasmids used in this study.

Designation	Genotype or description^a^	Source or reference
Strains		
*M. fulvus* 124B02	wild type strain, possessing pMF1 plasmid	3
*M. xanthus* DZ1	nonmotile, nonfruiting, dispersed-growing	D. R. Zusman, University of California, Berkeley
*E. coli* DH5α	*sup*E44, *Δlac*U169 (ϕ80*lacZ*ΔM15), *hsd*R17, *rec*A1, *end*A1, *gyr*A96, *thi*-1, *rel*A1	Life Technologies Inc.
Plasmids		
pMF1	a cryptic plasmid from *M. fulvus* 124B02, 18.634 kb	3
pSQ37	Amp^r^, pSP72 with the insertion of a 10.8-kb *EcoR*I fragment of pMF1(16382-8545), 13.2 kb	This study
pSQ38	Amp^r^, pSP72 with the insertion of a 7.8-kb *EcoR*I fragment of pMF1(8545-16382), 10.2 kb	This study
pZJY41	shuttle plasmid from a DZ1 transformant of pZJY7	3
pXS1	pZJY41 with the insertion of a 3.8-kb *Sac*II fragment of pSQ37(3680-7410 of pMF1)	This study
pXS2	pZJY41 with the insertion of a 4.9-kb *Sac*II fragment of pSQ37(17270-3680 of pMF1)	This study
pXS3	pZJY41 with the insertion of a 3.2-kb *EcoR*I-*Nsi*I fragment of pSQ37(16382-971 of pMF1)	This study
pXS4	pZJY41 with the insertion of a 7.5-kb *Nsi*I-*EcoR*I fragment of pSQ37 (971-8545 of pMF1)	This study
pXS5	pZJY41 with the insertion of a 3.7-kb *EcoR*I-*Pvu*I fragment of pSQ38 (8525-12280 of pMF1)	This study
pXS6	pZJY41 with the insertion of a 4.1-kb *Pvu*I-*EcoR*I fragment of pSQ38 (12280-16382 of pMF1)	This study
pXS7	pZJY41 with the insertion of a 1.5-kb *EcoR*I-*Bst*EII fragment of pSQ38 (8545-10132 of pMF1)	This study
pXS8	pZJY41 with the insertion of a 6.2-kb *Bst*EII- *EcoR*I fragment of pSQ38 (10132-16382 of pMF1)	This study
pXS11	pZJY41 with the insertion of a 2.5-kb fragment from pMF1(17242-50)	This study
pXS12	pZJY41 with the insertion of a 2.5-kb fragment from pMF1(17242-1136)	This study
pXS13	Ampr, Kmr, pXS11 with the deletion of a *pMF1.21*	This study
pXS14	Ampr, Kmr, pXS11 with the deletion of a *pMF1.22*	This study
pXS15	Ampr, Kmr, pXS11 with the deletion of a *pMF1.23*	This study
pXS16	Ampr, Kmr, pXS11 with the deletion of *pMF1.22* and *pMF1.23*	This study
pET15b	Expression vector	Novagen
p15b21	Ampr, *pMF1.21* cloned into pET15b	This study
P15b23	Ampr, *pMF1.23* cloned into pET15b	This study

Depending on the inserted fragment, the inheritance stability of these plasmids in DZ1 varied greatly (Fig. S1). Compared to pZJY41, the plasmids pXS2 (containing nt 17270 to 3680 of pMF1) and pXS3 (containing nt 16382 to 971) were highly stable in DZ1. In the absence of the selective antibiotic kanamycin, colonies harboring the pXS3 and pXS2 plasmids comprised respectively 86% and 60% of the bacterial population after 48 h (approximately 12 generations; the double time of DZ1 is 4 h, ref 40) and 77% and 57% of the bacterial population after 120 h (approximately 30 generations), whereas the pZJY41-carrying colonies comprised <25% of the total population after 48 h and <10% after 120 h. These results suggest that the inserted fragments in pXS3 and pXS2 contained the partitioning system of pMF1.

### Structural characteristics of the partitioning system of pMF1

**Figure 1:**
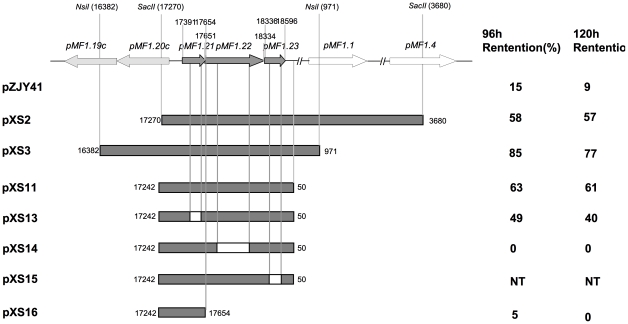
Schematic representation of pXS2, pXS3 and the in-frame deletion regions in *pMF1.21*, *pMF1.22* and *pMF1.23* and their influence on the stability of the plasmids. The white portion within the gray bar stands for the deletion of the corresponding genes in pXS13, pXS14 and pXS15. The corresponding stability of these plasmids in *M. xanthus* after growth for 96 h and 120 h without selective pressure is indicated on the right. NT, not tested.

The common region of the fragments inserted in plasmids pXS2 and pXS3 was identified to be nt 17270 to 971 of pMF1, which contained three ORFs, *pMF1.21*, *pMF1.22* and *pMF1.23* (Fig. 1). It is noted that although the presence of *pMF1.20* gave a further increase to the stability of plasmid (pXS2 vs pXS3, 40% improved stabilization), the gene has a reverse transcription direction to the above three gene operon, and was thus probably not included in the partitioning system. However, *pMF1.20* or together with *pMF1.19*, may play a role on plasmid maintenance. The products of both *pMF1.19* and *pMF1.20* have high similarities to *M. xanthus* DK1622 chromosome genes MXAN6992 and MXAN 6330 [3].

**Figure 2:**
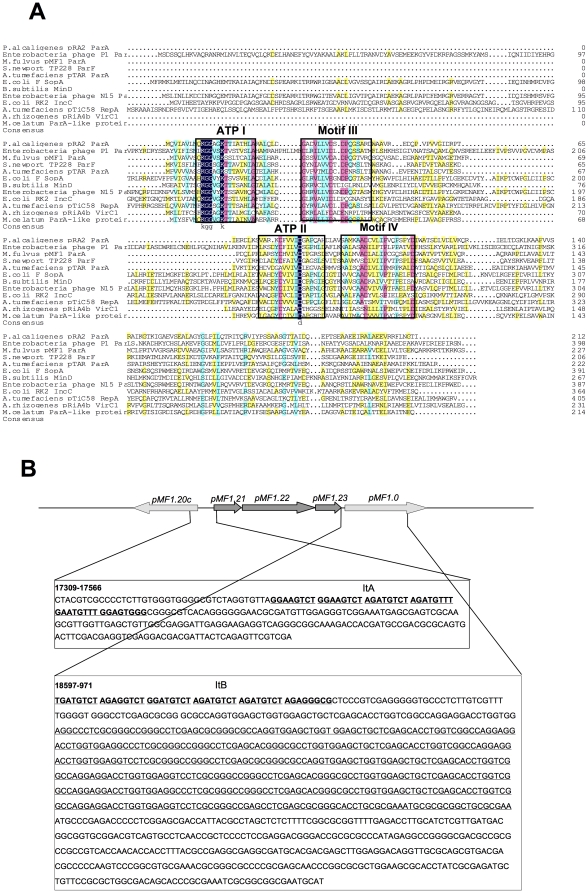
Genetic organization of the *par* loci of pMF1. (A) Alignment of the amino acid sequences encoded by pMF1 *pMF1.22* and other *parA* genes of other *par* systems. Database accession numbers: *Myxocuccus fulvus* pMF1 ParA, ABX46805; *Agrobacterium tumefaciens* pTiC58 RepA, AAF00012; *A. tumefaciens* pTAR ParA, P07175; *A. rhizogenes* pRiA4b VirC1, P05682; *Bacilus subtilis* MinD, Q01464; *E. coli* F SopA, P08866; *E. coli* RK2 IncC, P07673; *Enterobacteria* phage N15 ParA, AA19064; *Enterobacteria* phage P1 ParA, P07620; *Mycobacterium celatum* pCLP ParA-like protein, AAD42964; *Pseudomonas alcaligenes* pRA2 ParA, AAD40334; and *Salmonella newport* TP228 ParF, AAF74217. The framed boxes labeled ATP I and ATP II represent the ATPase motif regions, while those marked Motif III and Motif IV represent the additional conserved motifs in ParA family ATPases. The consensus amino acids are shaded. This figure was adapted from Motallebi-Vershareh *et al*, 1990 [32]. (B) The genes *pMF1.19* to *pMF1.1* of pMF1. The arrows indicate the coding genes and their transcriptional orientation. The three black arrows are the *pMF1.21* through *pMF1.23* genes in the pMF1 *par* operon. Nucleotide sequences of the region upstream (nt 17309 to 17566) and downstream (nt 18596 to 971) of the *par* operon are shown. The iterons ItA and ItB are bold and underlined and the “long repeats” are underlined.

The product of *pMF1.22* has extensive homology to the ParA family of ATPases, with the highest similarity of 56% to the amino acid sequence of the *Sphingobium japonicum* uncharacterized ParA-like protein. Amino acid sequence alignment analysis showed that the pMF1.22 protein contains the four conserved motifs of type I ParA proteins (Fig. 2A), which belong to the Walker-type ATPases [4]. It was thus suggested that the unknown pMF1.22 is a ParA-like protein, and the *pMF1.22* gene was designated *parA*. To determine the function of this gene *in vitro*, we attempted to express the *pMF1.22* gene in *E. coli* using several different hosts and vectors but did not obtain soluble product (data not shown). However, following genetic analyses indicated that the *pMF1.22* gene was essential for stably partitioning of plasmids in *Myxococcus* cells (see below). The other two ORFs, *pMF1.21* and *pMF1.23*, had no significant similarity to any entries in GenBank. These three ORFs were suggested to be the potential components of the partitioning system of the plasmid pMF1. The neighboring upstream and downstream sequences of these three genes contained some imperfect repeats, designated iterons ItA and ItB, respectively. In addition, there were several copies of “long repeats” (underlined) immediately downstream of the ItB sequence (Fig. 2B).

**Figure 3:**
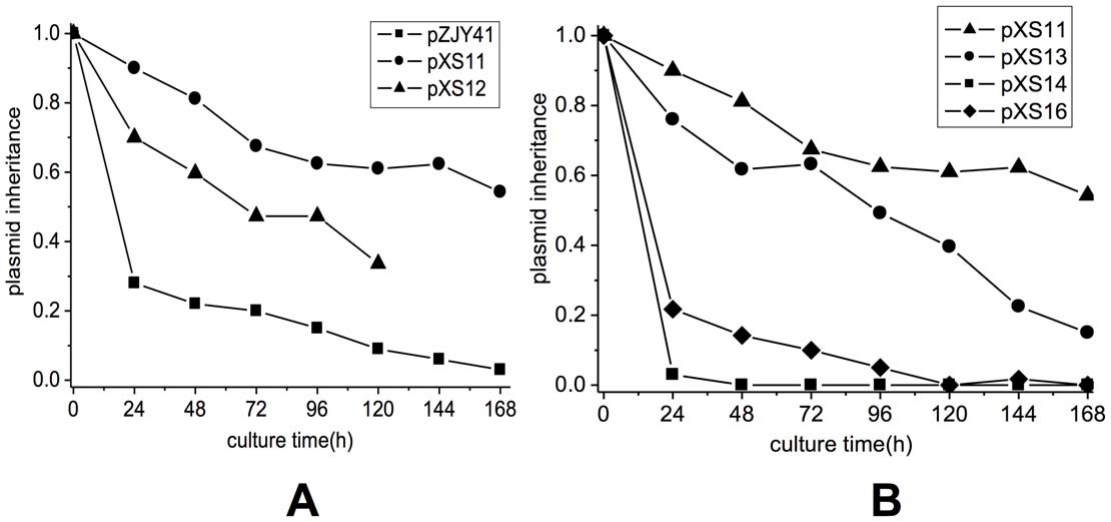
The inheritance stability of the plasmids carrying different fragments of pMF1 in *M. xanthus* DZ1 in the absence of the selective antibiotic kanamycin (panels A and B). The cultures grown in CTT medium were analyzed for plasmid retention at 24 h intervals until 168 h. The data presented are the average of two independent experiments.

To identify the components of the hypothesized pMF1 partitioning system, we constructed plasmids pXS11 and pXS12 carrying parts of the above common region; pXS11 contained all three genes and the ItA and ItB iterons, while pXS12 contained the same components and the “long repeat” downstream of ItB (Fig. 2B). Similar to pXS2 and pXS3, the plasmids pXS11 and pXS12 were also highly inheritably stable in *M. xanthus* DZ1 (Fig. 3A). After 120 h of incubation without antibiotic selection, plasmids pXS11 and pXS12 were retained in 61% and 34% of the bacterial population. The “long repeat” in pXS12 seemed to have small negative effects on plasmid stabilization. We concluded that the fragment in pXS11 containing the *pMF1.21*, *pMF1.22* and *pMF1.23* ORFs and the ItA and ItB iterons constituted the partitioning cassette of pMF1 (Fig. 1).

### Essentiality of *pMF1.21*, *pMF1.22* and *pMF1.23* in plasmid partitioning in *Myxococcus*

Bioinformatics analysis suggested that the *pMF1.21-pMF1.23* genes were in the same transcriptional unit, which was further confirmed using reverse transcription PCR of the cDNA from *M. xanthus* DZ1 transformants of pXS11 using the primer pair of parC-up and parB-down (Table 2; detailed results not shown). This suggested that the pMF1 partitioning system contained three genes. However, the partitioning system of a plasmid typically contains two genes, *parA* and *parB*. To determine the essentiality of the three genes for plasmid segregation, we made in-frame deletion mutations in *pMF1.21*, *pMF1.22* and *pMF1.23*, respectively, producing pXS13, pXS14 and pXS15 (Fig. 1). These plasmids were then transferred into the *M. xanthus* DZ1 strain, respectively. The pXS13 and pXS14 plasmids extracted from the *M. xanthus* DZ1 transformants were structurally stable, while pXS15 yielded only one positive clone, which, however, became smaller than the original size. This result suggested that the *pMF1.23* gene played an essential role in the maintenance of the plasmid and the mutation in *pMF1.23* was lethal to the *Myxococcus* cells that harbored the plasmid.

**Table 2:** Oligonucleotides used in this study.

Primer	Sequence	pMF1 coordinates
par-up	5′ccggatccCGCATCGGGTGAGCGTAGAG3′	17242-17261
par-down1	5′ccggaattcCCCACCCCAAAACGACAAGAGG3′	50-29
par-down2	5′ccggaattcTCGCCGCAGCTTCAGCCCTC3′	1136-1117
*parCmu-up2	5′CGACTCGCTCATTTCCGACCC3′	17427-17447
*parCmu-down1	5′GGGTCGGAAATGAGCGAGTCGATGGTCGCGCAGGCGGTGGAG3′	17427-17447 17610-17630
*parAmu-up2	5′TTCGCCAGCTCCACCGCCTGC3′	17618-17638
*parAmu-down1	5′GCAGGCGGTGGAGCTGGCGAAGTCCTCTCCGCCTCAACCACC3′	17618-17638 18146-18166
*parBmu-up2	5′AACCTCGGCCGGCGGGGCACG3′	18372-18392
*parBmu-down1	5′CGTGCCCCGCCGGCCGAGGTTCAAATTGCCGAGGAGTTGTTCGAC3′	18372-18392 18555-18578
**parC-u:	5′gggaattc CATATGGGCGGGCGTCACAGGG3′	17392-17409
**parC-d:	5′cgcggatcc TCATCGGCCCGCCTTCTTCGC3′	17634-17654
**parB-u:	5′gggaattc CATATGCGTGCCCCGCCGGCCGA3′	17370-17388
**parB-d:	5′cgcggatcc CTACTTGGGGAGATACTTGTCGA3′	17574-17596
DupupBiotin	5′CGCATCGGGTGAGCGTAGAG3′	17242-17261
Dupdo	5′CGACTCGCTCATTTCCGACCC3′	17428-17447
ItA	5′GGAAGTCTGGAAGTCTAGATGTCTAGATGTTTGAATGTTTGGAGTGGG3′	17348-17395
ItB	5′GTGATGTCTAGAGGTCTGGATGTCTAGATGTCTAGATGTCT3′	18596-3
ItC	5′AGACATCTGGAAGTCTGGATGTC3′	13514-13537
ItD	5′AGAAGTCCAGACATCTGGACATTCAGAAGTCTAGACATCTGGATGTCCAGATGGGG 3′	14102-14157
ItE	5′AAGTGTCTAGATGTCTAGATGTCT3′	14947-14970
ItF	5′AGAGGTCTAGGTGTCTGGAAGTCTGGAGGTCTGGATGTC3′	15863-15902
***parC-up	5′GGAAGAGGTCAGGGCGGCAAAG3′	17480-17501
***parC-down	5′GCCTTCTTCGCCAGCTCCAC3′	17625-17644
***parA-up	5′ATGATTGTCGCGGTCGTGTCCC3′	17651-17672
***parA-down	5′TCCGCATCCACCACCAGCACG3′	17743-17763
***parB-up	5′TCCGAGCGACCCTCAACTTG3′	18449-18468
***parB-down	5′CCTCGGCAATTTGGGAGTGTTC3′	18546-18567
16S-up	5′CGCCGTAAACGATGAGAA3′	
16S-down	5′TTGCGT CGAATTAAACCAC3′	

*primers used in gene deletion.

**primers used in gene expression in *E.coli.*

***primers used in quantitative PCR.

The inheritance of the pXS13, pXS14 and pXS16 (with the deletion of *pMF1.22* and *pMF1.23*) plasmids in DZ1 is shown in Fig. 3B. The plasmid pXS14 (*pMF1.22* deleted) had low stability without selective pressure. The retention of the plasmid was less than 5% after 6 generations (24 h) and 0% after 12 generations (48 h). Similar result was observed in pXS16 (*pMF1.22* and *pMF1.23* were both deleted) plasmid stability test, 0% after 42 generations (120 h) (Fig. 3B). The mutation in *pMF1.21* (pXS13) alone also decreased the inheritance stability of the plasmid. Retention of the pXS13 plasmid in DZ1 was 49% after 24 generations (96 h) and 40% after 42 generations (120 h), which was still higher than that of pZJY41 but lower than the intact *par*-containing plasmid pXS11. These results indicated that the three genes played different roles in stable plasmid partitioning. The *parA*-like gene (*pMF1.22*) and *pMF1.23* genes are suggested to be essential, whereas *pMF1.21* plays an accessory role for stably partitioning plasmids in *Myxococcus* cells.

### The DNA-binding protein of the *Myxococcus* pMF1 *par* system

**Figure 4:**
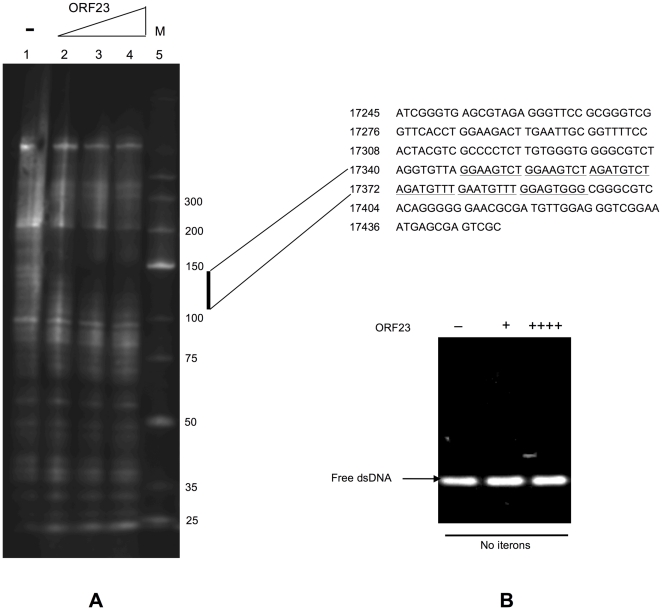
DNA-binding activity of ORF23. (A) DNase I footprinting analysis of the binding activity of ORF23 with the DNA fragment corresponding to nt 17242–17447 upstream of the *par* operon of pMF1. After incubation, the mixture was partially digested with DNase I and separated using denaturing gel electrophoresis. Lanes 1 to 4 contained 0, 250 ng, 1250 ng, and 3750 ng of ORF23, respectively. The ladder marker was in lane 5. The bar on the right indicates the tested DNA fragment containing the protected region of the iterons (underlined). (B) Electrophoretic mobility shift assays (EMSAs) showing the no DNA binding activity of ORF23 to non-iteron fragment nt 17395–17447. The concentration of labeled DNA used in each lane was 12 pmol. The amounts of purified ORF23 added in lanes 1 to 3 from left to right were 0 (−), 250 ng (+) and 1000 ng (++++), respectively.

**Figure 5:**
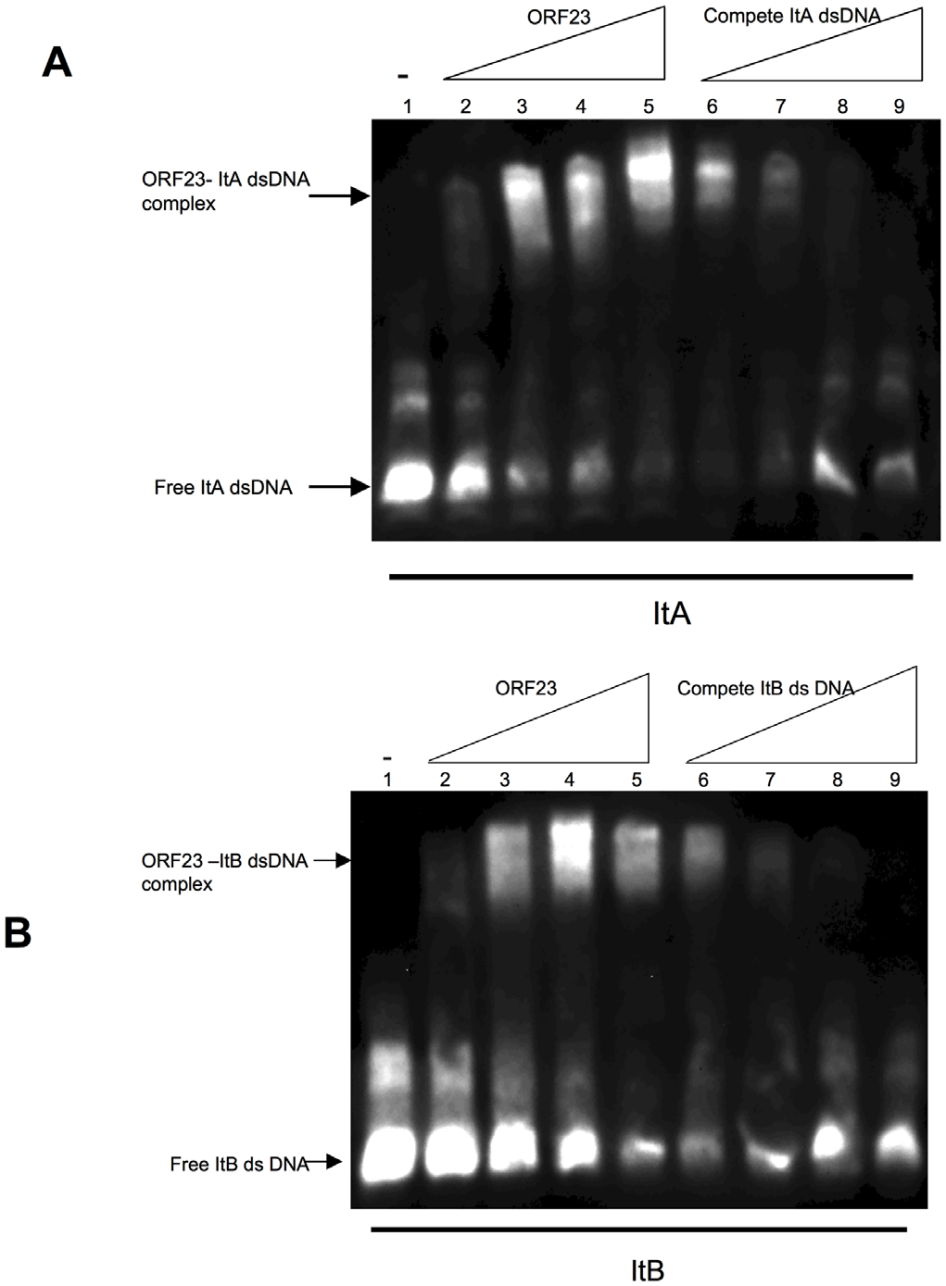
EMSAs showing DNA binding activity of ORF23 to the ItA sequence nt 17348–17395 (A) and the ItB sequence nt 18580-3 (B). The amount of purified ORF23 added in lanes 1 to 5 in (A) and (B) was 0 (−), 250 ng, 500 ng, 1000 ng, and 1500 ng, respectively. Increasing amounts of unlabeled DNA fragments (0, 0.8 pmol, 8 pmol, and 80 pmol) were incubated with 500 ng purified ORF23 protein in lanes 6 to 9 in (A) and (B), respectively.

In a plasmid partitioning system, a ParB or ParB-like component is required to recognize and bind to the corresponding centromere-like sequences to form a nucleoprotein complex, which localizes at a particular position in cell and moves into daughter cells for plasmid segregation when cell division occurs [21], [23], [24], [25], [26], [27]. To determine if one or both of the *pMF1.21* and *pMF1.23* products were responsible for DNA binding, we expressed these two genes separately in *E. coli* and obtained soluble proteins. A DNase I footprint assay was used to assess the binding abilities of the purified *pMF1.21* and *pMF1.23* proteins to DNA sequences containing the upstream fragment with the putative repeat ItA (nt 17348–17395). The fragment (206 bp, see Fig. 4A) was biotin-labeled at the 5′ end of the coding strand. After incubation with sufficient *pMF1.23* product, an approximately 100-bp sequence containing the putative iteron ItA (nt 17348 to 17447 of pMF1) was bound to the *pMF1.23* protein, and protected from DNase degradation (Fig. 4A). This 100-bp sequence was then divided into two fragments, one of which contained only the ItA sequence. These two fragments were labeled separately with biotin at the 3′ end to serve as probes for an electrophoretic mobility shift assay (EMSA). The *pMF1.23* protein bound to the ItA sequence (nt 17348–17395) (Fig. 5A) but not to the other fragment (nt 17396–17447) (Fig. 4B). The amount of the nucleoprotein complex increased with an increase in the concentration of the *pMF1.23* product, and the addition of unlabeled ItA fragment was competitive, reducing the amount of labeled complex. To exclude possible effects of His-tag on the binding activities [28], we further constructed His-tag free pMF1.23 protein. Compared to the pMF1.23 protein with a His-tag, the protein without a His-tag had a similar binding affinity to ItA (Fig. S2), which suggested that the His-tag at the N-terminal did not affect the affinity of the protein. In contrast to pMF1.23, the pMF1.21 protein did not bind and thus did not protect either of the fragments (data not shown). The *pMF1.21* and *pMF1.23* genes were thus designated *parC* and *parB*, respectively. Because the product of the *pMF1.23* gene is 75 amino acids long, the pMF1 *par* system probably belongs to type Ib (the ParB proteins of type Ia partition systems are much larger than that of type Ib, see reference 4).

### Iteron sequences for plasmid partitioning and their distribution in pMF1

Because the sequences upstream and downstream in the *par* loci are both imperfect repeats (Fig. 3B), we investigated whether the repeat sequence (nt 18597-3) downstream of the *par* loci (ItB) was also a binding site for ParB. After labeling with biotin at the 3′ end, the probes were incubated with the *pMF1.23* protein (ParB). The *pMF1.23* product also bound to the ItB probe and formed DNA-protein complexes (Fig. 5B). Similarly, the amounts of the nucleoprotein complex increased with increasing *pMF1.23* product concentration, and the addition of unlabeled ItB fragment was competitive, reducing the amount of the labeled complex of the ItB probe.

**Figure 6:**
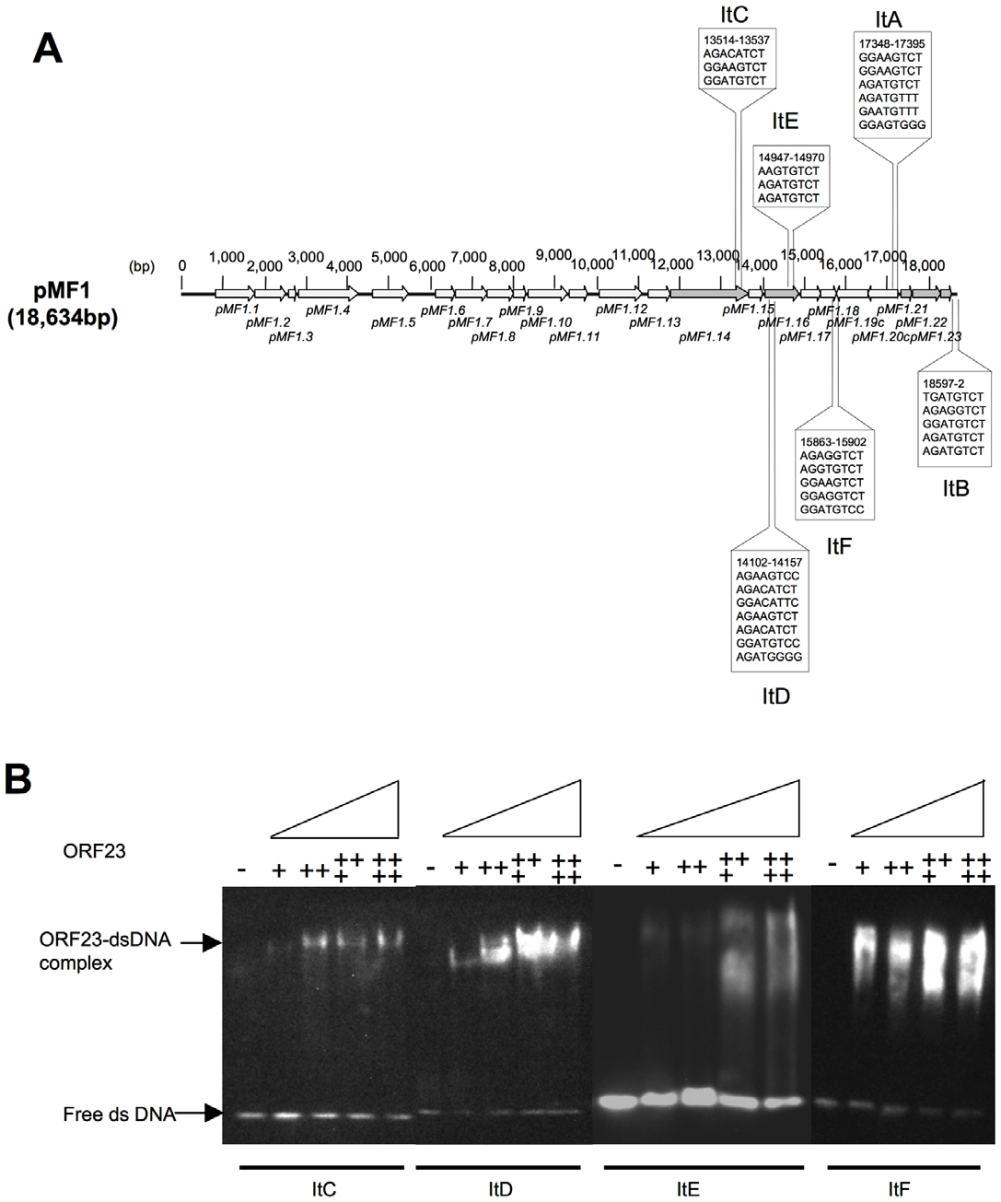
Iterons in the plasmid pMF1. (A) Distribution of predicted iteron sequences in the plasmid pMF1. (B) The binding activity of ORF23 to ItC, ItD, ItE and ItF. The substrate DNA used is indicated at the bottom of the figure. The amounts of ORF23 added to each lane are indicated at the top of the figure as follows: 0 (−), 250 ng (+), 500 ng (++), 1000 ng (+++), 1500 ng (++++).

Compared to other plasmid partitioning systems, the iteron copy number in the pMF1 *par* operon was much lower (upstream ItA and downstream ItB). Further bioinformatics analysis of the pMF1 sequence revealed several additional centromere-like sites dispersed mainly between the replication-associated region (*pMF1.14*) and the partition loci, from nt 12511 to 3 of pMF1, including in the *pMF1.14* and *pMF1.16* ORFs (Fig. 6A). These sites, designated ItC, ItD, ItE, and ItF, as well as ItA and ItB contain repeat units similar to the motif of AGATGTCT, which is an imperfect complement repeat itself. The EMSAs indicated that ItC, ItD, ItE, and ItF were also specifically bound by the *pMF1.23* product (Fig. 6B). Therefore, the iterons of pMF1 are distributed not only near the partitioning loci but also throughout the plasmid.

### Autogenous regulation of expression of the *par* loci in pMF1

**Figure 7:**
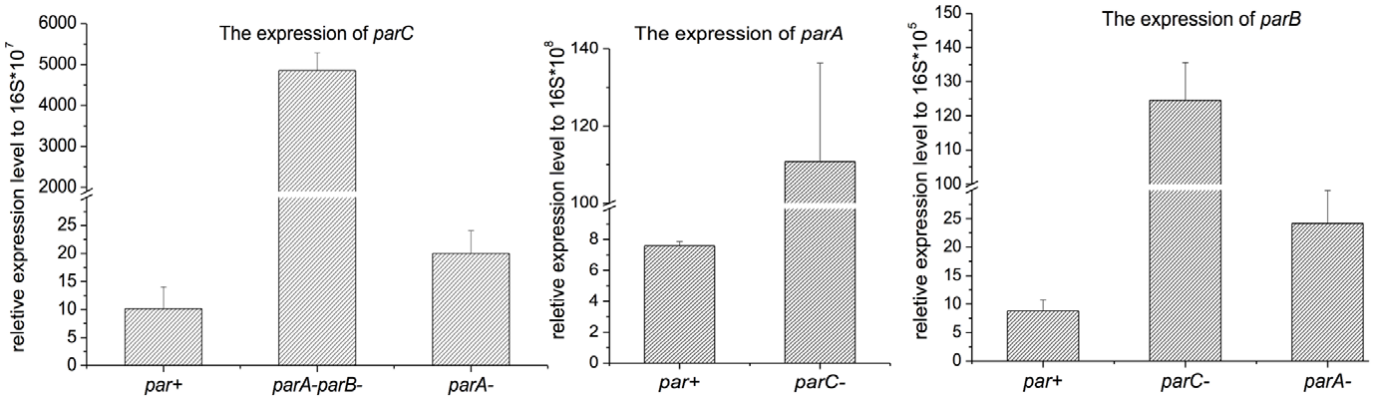
Quantitative PCR analysis. The gene expression of *parC*, *parA* and *parB* of the deletion mutants of pXS13 (*parC^−^*), pXS14 (*parA^−^*) and pXS16 (*parA^−^parB^−^*) relative to that of the 16S gene and compared to that of the transformant of pXS11 (*par^+^*).

The *par* system is typically autogenously regulated by its products. In type Ia *par* loci, ParA is the regulator, while in type Ib and type II, the ParB protein regulates the expression of the partition-related proteins [4]. Because pXS15 (*pMF1.23* mutated) is lethal for DZ1 cells harboring the plasmid, we constructed a *par* mutant containing only the promoter and the *parC* gene (*pMF1.21*) of the pMF1 *par* loci in the pZJY41 plasmid (designated pXS16) to determine the regulation pattern of the pMF1 *par* loci. Before quantify the expression profile, we assessed the copy numbers of the plasmids pZJY41, pXS11, pXS13 (*parC* ^−^), pXS14 (*parA* ^−^), and pXS16 (*parA^−^parB* ^−^) by comparing the fluorescence intensities of the bands under UV light, which showed that the copy numbers of pXS11, pXS13 and pXS14 were similar to that of pZJY41, 10 to ∼20 in *Myxococcus* DZ1 [3]; whereas the plasmid pXS16 was some lower (about half the number of other plasmids from the band density on electrophoretic gel) (Fig. S3). Then the gene expression profile of pXS13 (*parC* ^−^), pXS14 (*parA* ^−^), and pXS16 (*parA^−^parB* ^−^) was compared with that of pXS11 (*par* ^+^) using quantitative RT-PCR (Fig. 7). The results revealed that the expression of *parB* and *parC* in pXS14 (*parA* ^−^) was almost equivalent to that of pXS11 (*par* ^+^) in the DZ1 strain, which indicates that the ParA protein did not regulate gene expression in the *par* loci. However, the expression of the *parC* gene in the *parA^−^parB^−^* mutant (pXS16) was greatly increased, at least 200 times higher than that in pXS11. Therefore, the *pMF1.23* product (ParB) played an essential role in regulating the promoter activity of the *par* loci. This autogenous regulation pattern of pMF1 *par* loci is consistent with that of a type Ib partitioning system [4].

### Function of the ParC protein

**Figure 8:**
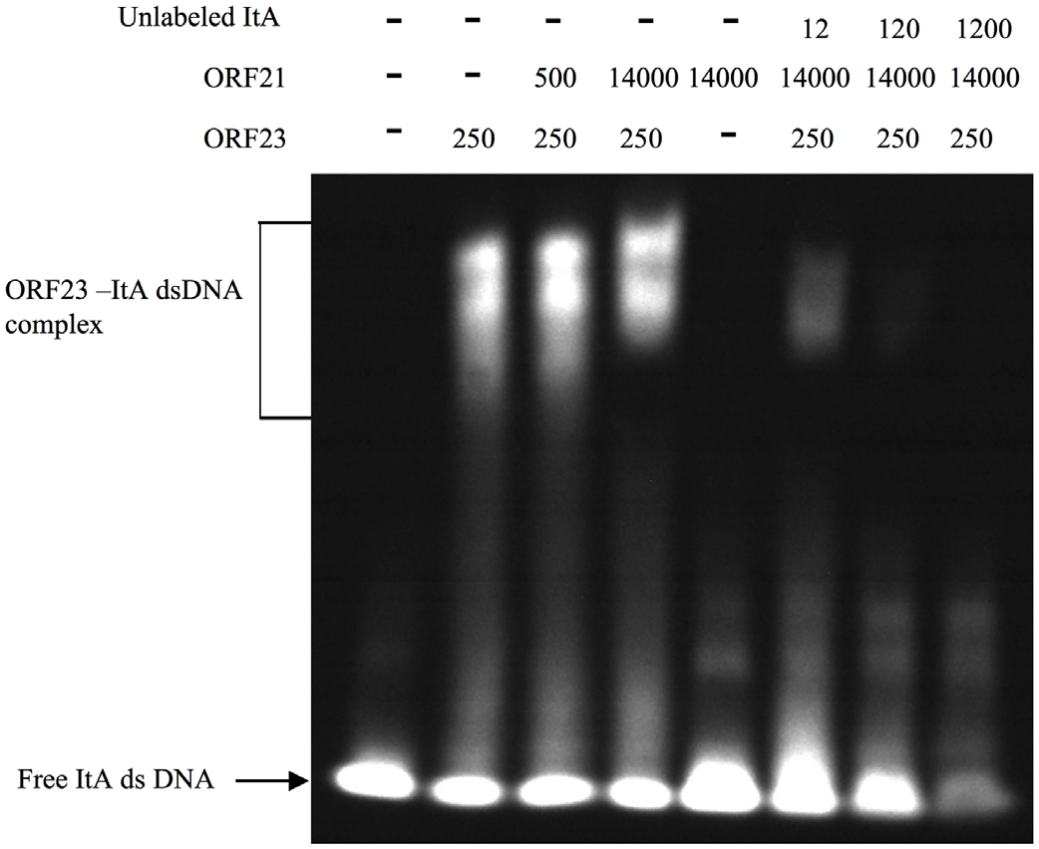
Binding of purified ORF21 and ORF23 to the ItA fragment. ItA was incubated with purified ORF23 and/or ORF21, the amounts of which are described at the top of the figure (ng). Indicated amounts of unlabeled ItA (pmol) was added to show the competition. The concentration of labeled DNA used in each lane was 12 pmol.

Compared to typical *par* systems, the pMF1 *par* loci contained an additional gene *parC*. Although the *pMF1.21* product alone did not bind to the iteron fragments, the protein was able to enhance the DNA-binding activity of the *pMF1.23* product (Fig. 8). If the amount of the *pMF1.21* product was much higher than the *pMF1.23* product (Fig. 8, lane 4), the enhancement was accordingly much greater than that when the two proteins were almost equally expressed (lane 3). The expression of the *parA* and *parB* genes in the *parC* mutant (pXS13) was also significantly increased, approximately 15 times higher than that in pXS11 (Fig. 7). The ParC protein thus had a repressive effect on the promoter of the *par* loci but much lower than that of the ParB protein. The ParC protein appears to play an accessory role in the pMF1 partitioning system by not only enhancing the DNA-binding activity of ParB but also regulating gene expression of the *par* loci.

## Discussion

Plasmid partitioning systems typically contain two genes encoding a *trans*-acting protein pair ParA/ParB and *cis*-acting iteron sites upstream and/or downstream of these two genes [4], [27], [29]. Represented by P1 ParA, the type Ia ParA contains an N-terminal DNA–binding domain, which plays an autoregulation role for the transcription of operon; whereas the type Ib, represented by pTAR, TP228, pB171 and pSM19035, encodes a ParA protein of 192–308aa, lacking the N-terminal DNA–binding domain [4], [6], [20], [21], [22], [23]. In the type Ib *par* system, the ParB-like protein serves as the autogenous regulator of the *par* operon by repressing the transcription of the *par* genes. The partitioning system of *Myxococcus* pMF1, described in this paper, contains three coding genes, *pMF1.21* (*parC*), *pMF1.22* (*parA*) and *pMF1.23* (*parB*), thus designated *parCAB*. Although the potential segregation-related region of *Pseudomonas alcaligenes* pRA2 also contains three ORFs, the *parC* is not required for plasmid stability and is likely not a coding region [26]. The three genes described in this paper all play roles in plasmid segregation. In the pMF1 *parCAB* system, the ParB protein is able to specifically bind to the iteron sites, which are not only distributed upstream and downstream of the operon but also dispersed between the replication-associated region and the *par* loci. Disperse centromere-like sites have also been found in the linear prophage N15 [30], the plasmid RK2 [31], [32], and streptococcal plasmid pSM19035 [20]. In the pMF1 *par* system, six centromere-like sites were identified. Although more stable than pZJY41, the pXS11 plasmid in *M. xanthus* DZ1 did not reach a similar high heritable stability as other *par* loci-constructed plasmids, probably because the *par* loci fragment in the plasmid contained only two centromere-like sites (upstream ItA and downstream ItB of the operon). In addition to its binding activity, type Ib ParB proteins also serve as autogenous regulators of the *par* loci by repressing the transcription of the *par* genes [4], [5], [26]. In plasmid pMF1, the ParB protein is essential for stability of the plasmid containing the *par* loci by binding to the iteron sequences for the partitioning process and regulating the expression of the *par* genes to avoid their over-expression. In the absence of the *parB* gene, the expression of the *parC* gene in *M. xanthus* DZ1 harboring the pXS16 plasmid (*parA^−^parB* ^−^) was more than 200-fold higher than that when the *parB* gene was present. Mutation of the pMF1 *parB* with the presence of the *parA* wild type allele is unsuccessful, which is probably due to that disruption of *parB* gene may lead to over-expression of ParA, which seems to be lethal to cells harboring pXS15 (*parB* ^−^). This is similar to the pRA2 partitioning system [26]. Binding of the ParB protein to the *cis*-acting sites functions not only in segregation of the plasmids containing the pMF1 *par* loci but also in regulating the expression of the operon, whereas ParA-like protein lacks the regulatory N-terminal. These results suggested that the *parCAB* system of pMF1 belonged to type Ib. In the type Ib plasmid pSM19035, Omega (ParB homologue) has multiple binding sites on the plasmid, serving as a global regulator [20]. The pMF1.23 also has multiple cognate sites on plasmid pMF1: ItA and ItB are close to the *parCAB* genes while the other centromere-like sites mainly localize between the replication–associated region (*pMF1.14*) [3] and the partition locus, from nt 12511 to 3 of pMF1. Furthermore, deleting *parA* and *parB* gene at the same time led a decrease in plasmid copy number (Fig. S3), whereas deleting *parA* alone did not have the same effect. These results probably hint potential regulating roles of pMF1.23 on plasmid replication and partition as a global regulator. The most unusual characteristic of the *par* loci of pMF1 is the presence of an additional gene, *parC* (*pMF1.21*). Although unable to bind to any of the DNA regions tested, ParC greatly enhanced the DNA-binding activity of ParB. ParB negatively regulated the *par* promoter, and qPCR results showed that ParC also repressed the expression of the *par* genes, although to a lesser extent than ParB. We hypothesize that the ParC protein probably binds to ParB-DNA complex to enhance the function of ParB and thus plays as a collaborator of ParB for its functions.

## Materials and Methods

### Plasmids and oligonucleotides

All the plasmids used in this study are described in Table 1, and the oligonucleotide sequences used in this study are given in Table 2.

### Bacterial strains and growth conditions

The myxobacterial strains were cultivated in CTT [33] medium at 30°C. The *E. coli* strains were incubated in Luria-Bertani (LB) medium at 37°C. When required, a final concentration of 100 µg/ml ampicillin and/or 40 µg/ml kanamycin was added to the solid or liquid media for selection.

### Cloning of the partition system of pMF1

pSQ37 and pSQ38 were subclones of pMF1 and contained the total sequence of pMF1. The boundaries of pMF1 fragments in pSQ37 and pSQ38 were the *EcoR*I sites. *EcoR*I/*EcoR*I fragments of pSQ37 and pSQ38 containing the pMF1 sequences were each digested with additional restriction enzyme. To preserve stability-associated region, we chose two different restriction enzyme pairs for each of the *EcoR*I/*EcoR*I fragments: *Nsi*I and *Sac*II were used with pSQ37, whereas *BstE*II and *Pvu*I were used with pSQ38. A library of 1.5 kb to 8 kb fragments was generated and inserted into the *EcoR*V site of pZJY41. *E. coli* DH5α was used as the plasmid host for the majority of cloning procedures in this study. Plasmid DNA was prepared, manipulated and transformed using standard procedures [34]. The recombinant plasmids containing various pMF1 fragments were electroporated into *M. xanthus* DZ1 according to the protocol described by Kashefi and Hartzell [35] and characterized using the plasmid stability assay described bellow.

### Plasmid stability assay

*M. xanthus* DZ1 cells carrying the plasmids to be tested for stability were grown to late exponential phase in CTT medium supplemented with 40 µg/ml kanamycin at 30°C. The culture was considered generation 0 and diluted 1∶25 in fresh CTT liquid medium without antibiotics and grown at 30°C for 24 h. Then, serial dilutions of the culture were plated on solid CTT medium without antibiotics. The dilutions and plating were repeated every 24 h, which represented an interval of approximately six generations. After each round, 120 single colonies from the plates were patched onto fresh CTT medium with and without kanamycin, and plasmid stability was measured as the percentage of antibiotic-resistant clones.

### Protein expression and purification

The coding regions of *pMF1.21* and *pMF1.23* were cloned under the control of the T7 promoter of pET-15b (Novagen) and recombinant proteins with a His_6_-tag were expressed in *E. coli* BL21 (DE3) after induction with 1 mM isopropyl-β-D-thiogalactopyranoside (IPTG). Soluble proteins were purified on a Ni-agarose column according to the manufacturer's protocols (Qiagen). Proteins used in EMSAs and DNase I footprint assays were at least 90% pure based on sodium dodecyl sulfate-polyacrylamide gel estimates, according to the method of Laemmli [36].

### Electrophoretic mobility shift assay (EMSA)

The DNA oligonucleotides were synthesized by Sangon Biotech Company (Shanghai) and labeled with biotin at the 3′ ends using the Biotin 3′ End DNA Labeling Kit (Pierce). Complementary biotin-labeled oligonucleotides were annealed as probes for DNA binding. The DNA probe (12 pmol) containing the iteron repeat fragment was incubated with the indicated amounts of ORF21, ORF23, or both purified protein preparations in reaction buffer (12 mM HEPES, 4 mM Tris-HCl, pH 8.0, 60 mM KCl, 5 mM MgCl_2_, 0.1 mM EDTA, pH 8.0, 1 µg poly(dI-dC) and 2% glycerol) for 30 min at 30°C. Unlabeled competitor DNA fragments were also used as indicated. Samples were loaded on a 5% native polyacrylamide gel and electrophoresed at 10 mA for 3 h. DNA was electrophoretically transferred to a nylon membrane, and the biotin-labeled nucleic acids were detected using the Chemiluminescent Nucleic Acid Detection Module (Pierce), according to the manufacturer's instructions.

### DNase I footprinting assay

The DNA fragment from nt17242 to 17447 of the pMF1 plasmid labeled with biotin at only the 5′ end of the coding strand was PCR-amplified using the primers DupupBio and Dupdo. The DNA probe was incubated with the indicated amounts of purified ORF23 in footprinting buffer containing HEMG buffer (50 mM EDTA, pH 8.0, 50 mM KCl, 12.5 mM HEPES pH 7.6, 6 mM MgCl_2_, 5% glycerol), 20 mM HEPES pH7.6 and 5 µg poly(dI-dC) in a final volume of 50 µl for 30 min at 30°C followed by adding 50 µl Ca/Mg Solution (5 mM CaCl_2_, 10 mM MgCl_2_) to the mixture. Then DNase I (Promega) and its buffer were added. After 3-min digestion, the stop solution was added. The resulting products were separated on an 8% polyacrylamide sequencing gel. DNA was electrophoretically transferred to a nylon membrane, and the biotin-labeled nucleic acids were detected following the same procedure as the EMSA described above.

### Quantitative real-time PCR analysis

*M. xanthus* DZ1 transformed using various plasmids was cultured on CTT solid medium containing 40 µg/ml kanamycin for 3–5 days and then inoculated in CTT liquid medium and incubated with shaking for 18 to 24 h at 30°C. The cells were collected, and the RNA was extracted immediately using the SV total RNA extraction kit (Promega) according to the manufacturer's instructions. Genomic DNA was eliminated using a DNA-free kit (ABI). The purified RNA was reverse transcribed to cDNA, and quantitative real-time PCR was performed in a BioRad sequence detection system with the iQ SYBR Green Supermix. 16S rRNA was used as the normalization signal [37]. The primers used for each gene were listed in Table 2.

## Supporting Information

Figure S1 **The inheritance stability of the plasmids constructed for isolation of the partition-associated region of plasmid pMF1 in** ***M. xanthus*** **DZ1 in the absence of the selective antibiotic kanamycin.** The data presented are the averages of two independent experiments.
(TIFF)

Figure S2 **EMSAs showing DNA binding activity of ORF23 with or without a His-tag to the ItA sequence nt 17348–17395. (A) Assays showing ORF23 with or without a His-tag specific binding to ItA.** Increasing amunts of ORF23 were incubated with ItA. The amount of the purified ORF23 with a His-tag added in lanes 1 to 5 or that without a His-tag added in lanes 6 to 10 was 0 (−), 250 ng, 500 ng, 1000 ng, and 1500 ng, respectively. **(B) Competition experiments showing ORF23 with or without a His-tag specific binding to the ItA.** The amounts of unlabeled DNA fragments (0, 0.8 pmol, 8 pmol, and 80 pmol) were incubated with 500 ng purified ORF23 protein with a His-tag in lanes 1 to 4 or that without a His-tag in lanes 5 to 8, respectively.
(TIFF)

Figure S3 **Agarose gel electrophoresis analysis of the pZJY41 derivates.** 6×10^8^ cells were used to extract the plasmid for each lane. Lane1, pZJY41; lane 2, pXS11; lane 3, pXS13, lane 4, pXS14; lane 5, pXS16; lane M, supercoiled DNA ladder markers. The size of each band is labeled on the right of the panel.
(TIFF)
