# Supplementary figures and images for: Characterization of the Partitioning System of Myxococcus Plasmid pMF1
Source: PLoS One. 2011 Dec 9;6(12):e28122. doi: 10.1371/journal.pone.0028122 (PMC3235114; doi:10.1371/journal.pone.0028122)

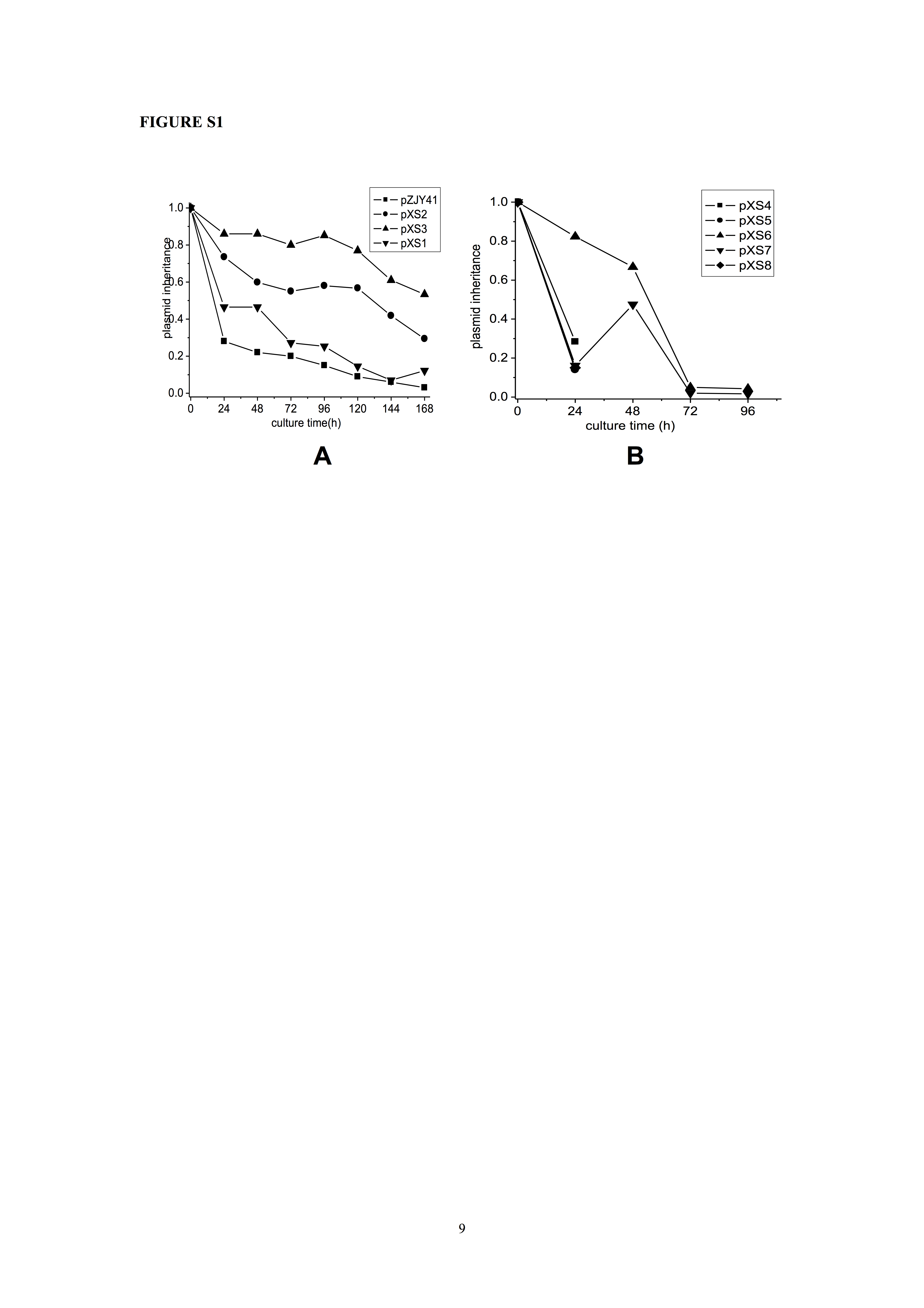

Supplement: Figure S1 — The inheritance stability of the plasmids constructed for isolation of the partition-associated region of plasmid pMF1 in M. xanthus DZ1 in the absence of the selective antibiotic kanamycin. The data presented are the averages of two independent experiments. (TIFF) [file pone.0028122.s001.tiff]

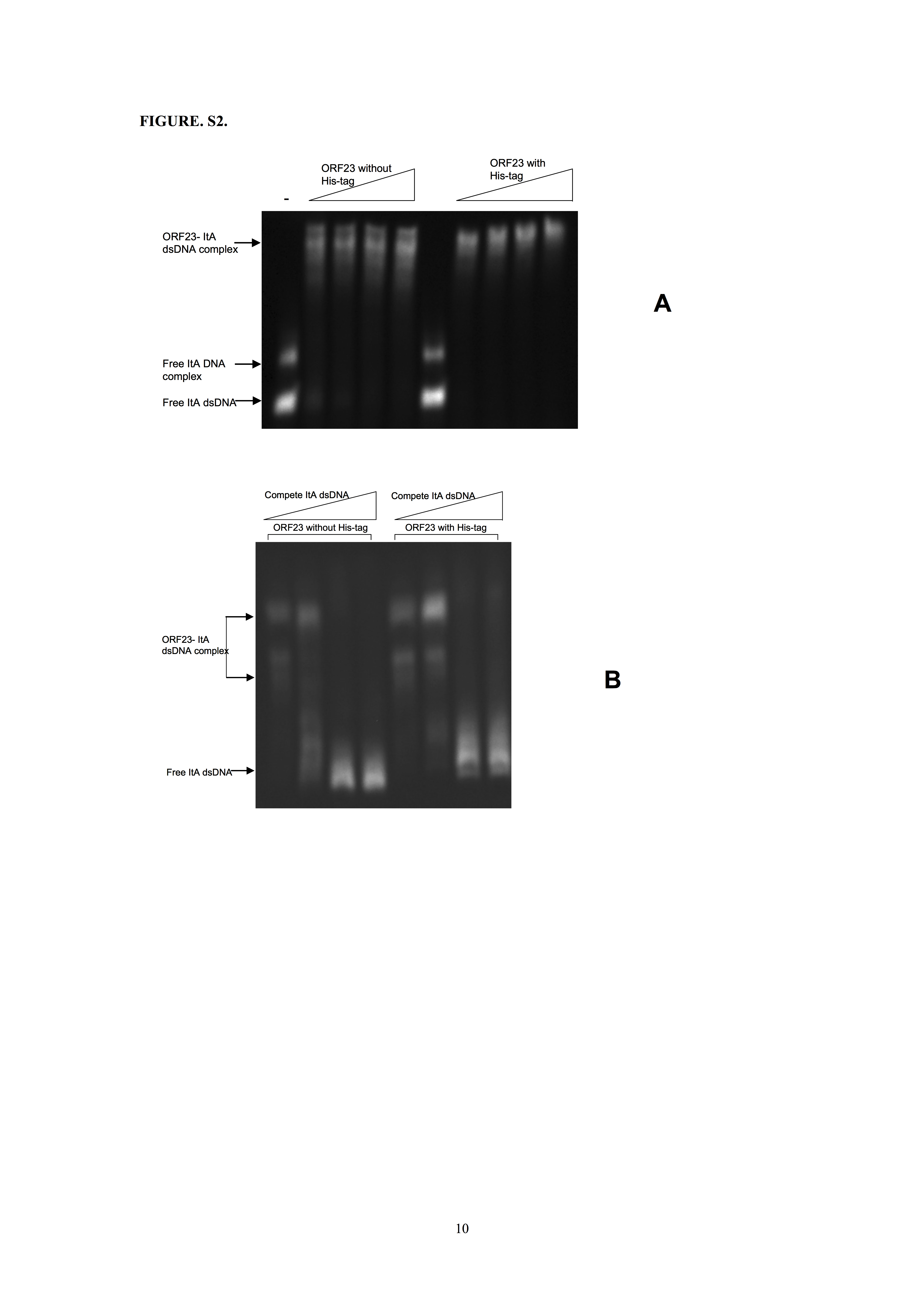

Supplement: Figure S2 — EMSAs showing DNA binding activity of ORF23 with or without a His-tag to the ItA sequence nt 17348–17395. (A) Assays showing ORF23 with or without a His-tag specific binding to ItA. Increasing amunts of ORF23 were incubated with ItA. The amount of the purified ORF23 with a His-tag added in lanes 1 to 5 or that without a His-tag added in lanes 6 to 10 was 0 (−), 250 ng, 500 ng, 1000 ng, and 1500 ng, respectively. (B) Competition experiments showing ORF23 with or without a His-tag specific binding to the ItA. The amounts of unlabeled DNA fragments (0, 0.8 pmol, 8 pmol, and 80 pmol) were incubated with 500 ng purified ORF23 protein with a His-tag in lanes 1 to 4 or that without a His-tag in lanes 5 to 8, respectively. (TIFF) [file pone.0028122.s002.tiff]

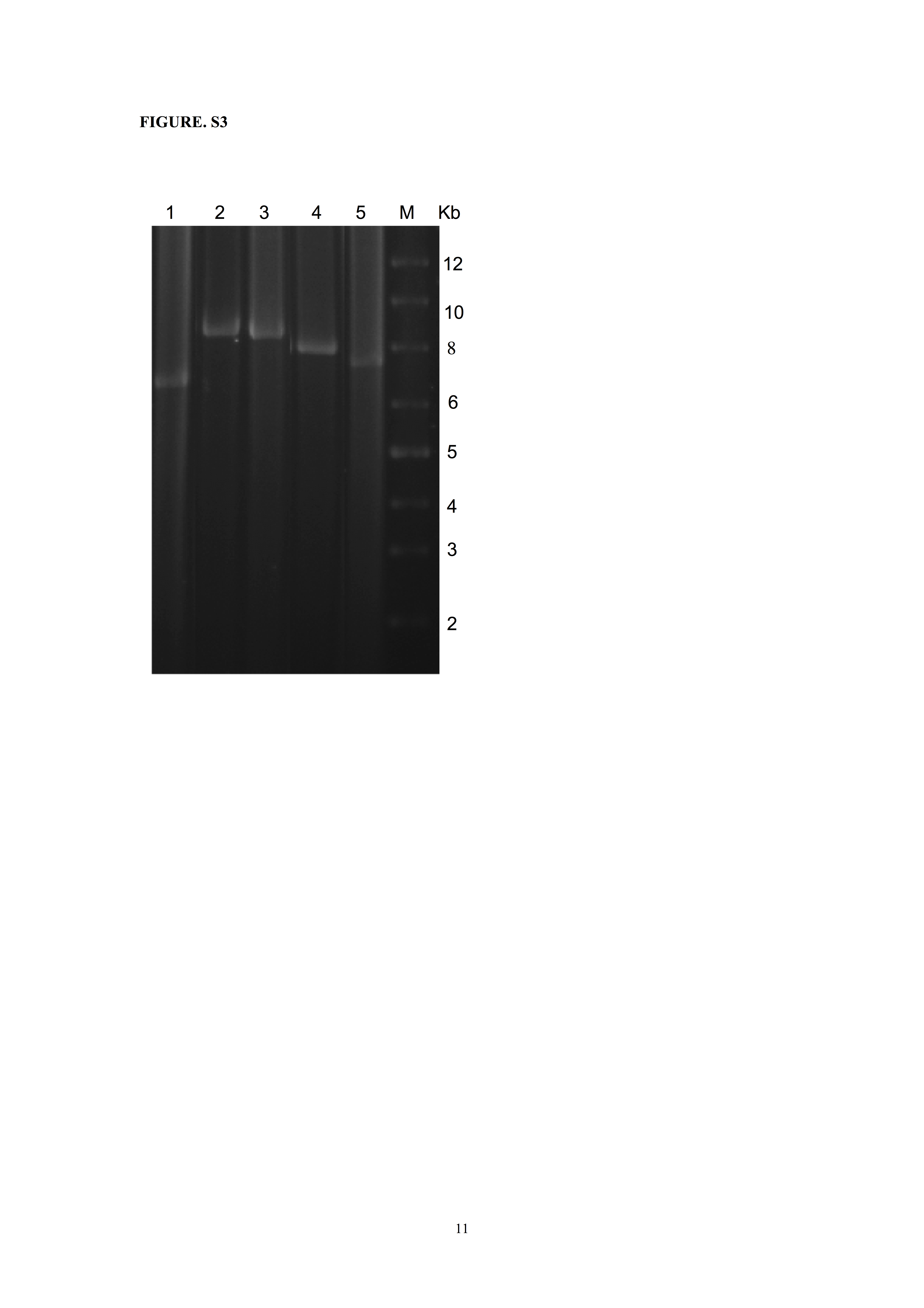

Supplement: Figure S3 — Agarose gel electrophoresis analysis of the pZJY41 derivates. 6×10^8^ cells were used to extract the plasmid for each lane. Lane1, pZJY41; lane 2, pXS11; lane 3, pXS13, lane 4, pXS14; lane 5, pXS16; lane M, supercoiled DNA ladder markers. The size of each band is labeled on the right of the panel. (TIFF) [file pone.0028122.s003.tiff]
